# Citizens and the state during crisis: Public authority, private behaviour and the Covid‐19 pandemic in France

**DOI:** 10.1111/1475-6765.12524

**Published:** 2022-03-26

**Authors:** CHRISTOPHER J. ANDERSON

**Affiliations:** ^1^ European Institute London School of Economics and Political Science London United Kingdom

**Keywords:** Covid‐19, compliance, legitimacy, France, public health

## Abstract

How do democratic states induce citizens to comply with government directives during times of acute crisis? Focusing on the onset of the Covid‐19 pandemic in France, I argue that the tools states use to activate adherence to public health advice have predictable and variable effects on citizens’ willingness to change their routine private behaviours, both because of variation in their levels of restrictiveness but also because of differences in people's political motivations to comply with them. Using data collected in March 2020, I show that people's reports of changes in their behavioural routines are affected by the signals governments send, how they send them and the level of enforcement. I find that a nationally televised speech by President Macron calling for cooperative behaviour and announcing new restrictions elevated people's willingness to comply. Moreover, while co‐partisanship with the incumbent government increased compliance reports before the President's primetime television address, presidential approval boosted reports of compliance after.

What is the capacity of states to make people do things they may not ordinarily do, especially in the face of an acute threat to the national community? How do citizens respond to the messages that public authorities send and the tools they deploy to produce compliance with government demands? Do people's political preferences and beliefs affect what they are willing to do to protect themselves and others? While these questions all have received renewed and urgent attention in the context of the ongoing Covid‐19 crisis, they also are long‐standing concerns for political scientists – they reach to the core of the state's powers to protect members of the national community from sudden and unpredictable threats to health and wellbeing.

To help shed new light on these questions, I examine the connections between what states do to activate compliance on one hand and how willing people are on the other to quickly adapt routine daily behaviours like travelling for work, maintaining physical distance in public, meeting friends or washing hands. Specifically, I consider the tools available to states to shape citizen behaviour, ranging from information provision about public health policies to ultimately enforcing protective measures. I argue that the state's use of these tools shapes people's behaviours, but also that citizens interpret instructions from government through their own lenses coloured by political preferences, competence judgments and perceptions of risk. Ultimately, then, state actions and citizen attitudes combine to shape people's willingness to adapt in times of crisis.

I test the argument with the help of a case study of France during the initial onset of the Covid‐19 pandemic. Specifically, I examine people's reactions to the policies implemented by the French government as well as the information communicated about them during March 2020, including a televised speech by French President Emmanuel Macron and the government's subsequent enforcement of public health measures. I find that the restrictiveness of government policies and the speech by President Macron were associated with shifts in reported and actual health‐related behaviours. Moreover, I investigated the individual‐level determinants of compliance reports. Taking advantage of the fortuitous timing of surveys conducted before and after the speech by President Macron and before and after the enforcement of a strict lockdown, I find that governing party supporters reported higher levels of compliance with public health messages before the President's dramatic primetime address. In contrast, satisfaction with the President's performance in office became a significant factor driving reports of compliance in the immediate aftermath of the speech. Ultimately, once the French state strictly enforced restrictions, political beliefs ceased to matter for reports of compliant behaviour.

Below, I first develop the argument, introduce the French case and specify expectations. I then evaluate several hypotheses about the connections between state actions and citizen behaviour, as well as between political beliefs and people's willingness to adjust their behaviour during times of acute crisis, with the help of reports of changes in behaviours, data on changes in people's actual mobility patterns, and individual‐level panel surveys. I conclude by discussing the limitations and implications of the study.

## Citizens and the State

Effective government requires voluntary cooperation and compliance from citizens across various domains of social, political and economic life.^1^ This is especially true during emergencies, such as the Covid‐19 pandemic in 2020, when governments around the world had to design and implement policies to ensure collective welfare on very short notice and under conditions of high uncertainty. Given the importance of citizen compliance for producing collectively beneficial outcomes, scholars for many decades have sought to understand people's willingness to cooperate.^2^ One stream of research has focused on what the state does or can do – the various levers of authority it can use to engender compliance. These take different forms, ranging from ‘softer’ tools in the form of information about risks or signals about desirable behaviours on one end all the way to ‘harder’ tools of coercion and punishment for non‐compliance on the other. In the context of the ongoing pandemic, public health messages or televised press briefings by public health officials are classic examples of such softer tools, while the deployment of police or military in the streets to enforce social distancing and control movement are obvious cases of intensely coercive instruments of authority.

Another explanation for why people comply is rooted in people's beliefs about the state – including, for example, whether they trust and approve of public authorities, how they view the demands put on them, or whether they look to state actors to help them orient themselves during times of uncertainty. Survey researchers in particular have concentrated on people's attitudes (e.g., citizens’ expressed trust in the system and officeholders, their willingness to obey the law, and their beliefs in the legitimacy of the state) as well as behavioural expressions of consent (e.g., whether and why people (dis)obey political authority) (e.g., Barnes et al., 1979; Klingemann & Fuchs, 1995; Levi & Stoker, 2000). In parallel, researchers taking a more rational approach have focused on people's willingness to incur the costs of solving social dilemmas. Given that acts of compliance are costly, with governments seeking to convert citizens’ private resources (e.g., time, money and sometimes even lives) into public ones, citizens’ incentives to comply are stronger when the benefits exceed the costs of doing so and when they hold positive attitudes toward political authorities (Anderson et al., 2020; Levi, 1988; Lieberman, 2009).^3^

Taken together, then, scholarship has painted a picture of compliant citizens that is shaped by what states do and what people believe.^4^ Yet, whether or how these drivers of compliant behaviour in fact operate during moments of acute crisis rather than during ordinary times is not a question that typically has been asked. Therefore, to better understand the connection between government actions and citizen responses and to test existing understandings of the relationship between citizens and the state, I examine people's willingness to comply with government demands with the help of a case study of France during the early weeks of the Covid‐19 pandemic in 2020. What did French public authorities do during the intense initial phase, as infections spread like wildfire? And how did citizens respond to the actions taken and the messages communicated by the Macron government? Specifically, how did they shape people's willingness to comply with public health directives regarding hygiene and social behaviours and restrictions placed on their physical movement?

## Compliance during the pandemic: The French case

The French government's effort to contain the virus began in late January 2020 and initially focused on travel restrictions for people coming to France from abroad as well as a public health information campaign at home.^5^ Yet, within a month's time, the government felt compelled to mandate the cancellation of all large‐scale public events, quickly followed by ever tighter restrictions on the scale and types of the events. By the middle of March, authorities had closed all schools and public transportation, non‐essential businesses and public services and workplaces. Thus, within just 7 weeks of the virus emerging as a public health threat, the French government had enacted a ban on all domestic travel,^6^ which was tightened even further within a week's time to include permitted movement only within 1 km (0.6 miles) of a person's residence, up to one hour per day, and only with a permission slip. These rules – unparalleled during peacetime in the nation's history – were backed by the state's visible exercise of its coercive powers.

While dramatic, France in many ways was emblematic of how most democratic governments reacted to the emerging threat posed by Covid‐19. During the early weeks and months, the spread of the virus typically went hand in hand with progressively more stringent restrictions on social interactions and movement. Among the challenges governments faced was the need to engineer changes in people's behaviours they could not easily observe directly or ones that people consider personal and private – for example, personal hygiene or rituals of social interaction.

On one hand, the task of changing non‐political behaviours is nothing new. States routinely seek to induce people to adapt their private and habitual behaviours to conform to directives and recommendations made by public authorities. At the same time, not since the Spanish flu of 1918 have citizens and governments had to grapple with a virus as contagious and as deadly. Moreover, neither governments nor citizens had the luxury of time: the threat was acute and posed an immediate and major public health emergency that required governments to come up with mitigation strategies overnight and people to adjust their behaviours on the fly.

Taken together, these challenges were far from trivial. Viewed from a theoretical perspective, the novelty and acuteness of the crisis could plausibly be expected to lessen or modify the effectiveness of government actions, messages and policies in activating citizen compliance – during such moments of radical uncertainty, citizens and experts alike find it difficult to assign probabilities to what might be the right policy decision and the right behavioural response (Kay & King, 2020). Consequently, compliance responses should be more difficult to engineer and therefore predict. Moreover, empirically speaking, we are only beginning to understand exactly how citizens reacted during the initial, most acute moment of the emerging crisis. To contribute to what we know, I therefore examine two sets of factors that can be expected to matter for shaping French citizens’ understandings and behaviours during the initial days of the pandemic: their exposure to signals and actions from the government, as well as their own understandings and interpretations of them.

### Exposure to public authority

Given the pervasive nature of the French government's information efforts during the early days of the pandemic, by the beginning of March, most French citizens had been exposed to messages about the risks of the virus and desirable behaviours to protect themselves and others. I expect these messages, along with ongoing reporting in the news media, to move reports of behaviours that could help reduce contagion so that, by the middle of March, reports of compliant behaviour among the French public should be at an elevated level.

Moreover, the French government became increasingly forceful in its approach to guide and control behaviour as the crisis escalated into early March. Most dramatically, on Monday, 16 March, President Emmanuel Macron spoke to the French nation in a primetime television address from the Élysée Palace. Using language usually reserved for times of war, he called on his compatriots to mobilize in the fight against an “invisible enemy” to protect the nation. As the COVID‐19 pandemic raged, he announced that France, one of the oldest democracies and a founding member of the European Union, would confine citizens to their homes, suspend municipal elections, give the government extraordinary powers to enforce the lockdown and close its borders to EU neighbours for a month.^7^ Moreover, he called on citizens to support the government's efforts:

My dear compatriots, I appreciate the impact of all these decisions on your lives. Giving up seeing your loved ones is a wrench; stopping your everyday activities and habits is very difficult. I'm asking you to make sacrifices to slow the epidemic.^8^

The most watched television program in the country's history,^9^ Macron's speech was a noteworthy intervention for *what* it communicated (new restrictions), *how* these were communicated (by drawing on the most potent symbols of authority available to the French state) and how many people it reached. I therefore hypothesize that the primetime speech to moved survey responses upward and beyond already elevated levels of compliance as the pandemic intensified. Only 1 week later, on 23 March, the government announced a further tightening of the strict home confinement rules, signalling the most severe shutdown of French public life during the pandemic, with police patrolling the streets and requiring residents to carry a signed form whenever they left their homes, as authorities were feverishly battling to contain one of the deadliest outbreaks of COVID‐19 in the world. As a result, I expect reports of compliance to be highest following the enforcement of the formal lockdown.

### Individual motivations to comply

While exposure to messages and actions taken by public authorities – like the dramatic speech by President Macron – provide citizens with information about risks as well as signals about rules regarding desirable behaviours in times of crisis, it is up to citizens to decide what to do with them. This means that people's own attitudes are likely to play a role in shaping reports of compliant behaviour as well, and in two ways. First, people's beliefs about the risks and threats posed by the virus can be expected to influence health‐related behaviours like social distancing or washing hands. Second, citizens interpret messages through the lens of their own preconceived notions about the messengers (public authorities), which, in turn, may affect how likely they are to listen to the advice and demands from them. Taken together, then, and following standard psychological theories of reasoned and planned behaviour, I thus expect the actions of governments to affect people's willingness to change behaviour directly as well as indirectly via their attitudes, and this willingness ultimately to produce the expected behaviour.

#### Attitudes about risk and threat

To begin, people's calculations of the costs and benefits associated with particular behaviours should affect their choices. On a personal level, people who are more risk averse when it comes to their personal health can be expected to take fewer risks, which is likely to translate – *inter alia* – into a greater willingness to take protective measures such as those publicized by governments via information campaigns during the early phase of the pandemic (e.g., washing hands, social distancing and so on).

In addition to personal risks, people can be expected to weigh the social risks of their behaviours. While the risk to the country initially was considered low – with a handful of documented infections and one confirmed death by mid‐February – by the middle of April, France had suffered a total of over 100,000 recorded infections and 17,000 official deaths from the virus. The speed with which the virus spread was reflected in a rapidly rising sense of threat among average citizens: while 30 per cent of the French public said they feared catching the virus on 10 March, that number more than doubled to 68 per cent within just 2 weeks’ time.^10^

As the virus took hold and perceptions of risk among the French public spiked, public discussion about the best ways to manage the pandemic threat was driven by two primary concerns around the threat to public health and the nation's economy. Restricting personal liberties and business activities meant an immediate threat to economic health, while keeping businesses, schools and public facilities open was seen as a potentially catastrophic threat to public health. Given the potential costs to the country's economic or physical health, I expect those who perceived more of a threat to report higher levels of compliance.

#### Political motivations to comply

While research in political behaviour seldom pays attention to private, habitual and ostensibly non‐political decisions, a rapidly growing stream of scholarship in economics and public policy has examined the ways in which the state can induce people to behave in ways that enhance individual and collective welfare. Commonly referred to under the heading of ‘nudges’, different strands of research have sought to investigate the effectiveness of non‐coercive policy tools for bringing about behaviours that are desirable from a collective welfare perspective.

Most relevant for purposes of this study is the work of scholars in behavioural public policy who have built on ideas from political psychology to examine how political predispositions like partisanship help explain decision making, and in particular how biases and heuristics shape citizens’ ‘processing of performance information provided by, or concerning, governments’ (for a review, see Moseley, 2020).^11^ Building on the literature on cue‐taking, which suggests that political beliefs act as a shortcut to credible information, the biases and heuristics that are most likely to matter and shape compliance during the pandemic are attitudes about political authorities. In moments of crisis and when the issues are technical, complex and confusing, citizens are especially likely to rely on cues, including source credibility, to determine whether to accept messages (Druckman, 2001; Gilens & Murakawa, 2002). Credibility requires that citizens believe a messenger possesses knowledge about relevant information and that they can be trusted to share that knowledge (Lupia & McCubbins, 1998).

Most prominently, in ordinary times, people's faith that messengers fulfil these conditions can be expected to stem from partisan attachments. Perhaps the most studied individual‐level political predisposition in political science, partisanship has been shown to affect a variety of cognitions, emotions and behaviours, including people's consumption choices or avoiding social situations and information that conflict with one's previously held beliefs (Huddy & Banker, 2017; Lelkes & Westwood, 2017; McConnell et al., 2018). Given that those who steer the ship of state represent partisan political ‘teams’ (Huddy et al. 2015), citizens whose partisanship matches that of the incumbent political authorities should be more likely to find information provided by them credible and conform with guidelines issued by them.^12^ Specifically, I expect supporters of the party of the President of the Republic (La République En Marche), which also held a large majority of seats in the French parliament at the time of the pandemic outbreak, to be more likely to report compliant behaviours.

In addition to partisanship, I expect evaluations of the current performance of public officials to shape people's willingness to adhere to government regulations. Specifically, citizens who are satisfied with the job public officials are doing should be more likely to find them to be credible sources of information and therefore report behaviour consistent with instructions from them, perhaps especially during moments of acute crisis when uncertainty is high and incumbents operate the most obvious levers of state power (see also Bol et al., 2020).^13^ In the French context, no public official stands out more than the President of the Republic, and I expect those who approve of the President's performance to be more likely to report compliance with government instructions.

While approval of government actors and partisan attachments are likely to be correlated empirically, they are distinct psychological constructs – with the former more performance‐based and volatile and the latter more reflective of political identities and stable. This also means that partisanship, as a long‐standing predisposition and habitual lens people use to assess sources and information than approval of governing authorities, should have a stronger effect on compliance reports prior to the unusual short‐term intervention produced by the Macron speech. In contrast, the staging and timing of the speech should remind citizens of the powers of the Presidency and public authorities, stimulating both a rally around the flag effect confirmed in other research (Bol et al., 2020; de Vries et al., 2021) as well as enhanced reports of compliance among those who approve of President Macron's handling of the crisis after the speech.

Although I expect both kinds of attitudes to shape how people select and interpret new political information and assess the credibility of the source providing it, there should be a natural limit to their power, depending on whether governments rely on voluntary adherence in order to achieve a collectively beneficial end or use force to compel obedience. As a consequence, the impact of political beliefs on compliance reports should be dynamic: they should be related to compliance only when the state has not yet formally invoked its authority and enforced restrictions on citizens’ behaviours. Once armed police are patrolling the streets, individual preferences should cease to matter altogether.

## Data and research design

I use data from several sources to examine the French public's reports of compliance during the early phase of the pandemic. Specifically, I begin by tracing the aggregate shifts in reported and actual behaviours before and after the speech by President Macron on 16 March, as well as before and after the subsequent lockdown on 23 March. I then examine the effects of the Macron speech and the lockdown on reports of compliance with the help of individual‐level survey data, followed by an analysis of the individual‐level determinants of compliance reports.

### Aggregate shifts in compliance

Did people's Covid 19‐related behaviours look different before and after 16 March and before and after 23 March? I analyzed the trajectories of state actions and citizen compliance with the help of data from several sources. First, I examined aggregate survey data to see if there were measurable differences in people's reported private behaviours during this crucial period. Alongside, I investigated whether an increase in the restrictiveness of government regulations preceded changes in reported and actual behaviours.

The survey data were collected in four separate surveys between 10 and 27 March, 2020.^14^ Respondents were asked how often they had taken measures to protect themselves or others from coronavirus, including touching objects in public, avoiding crowded areas, working outside the home and washing hands and using hand sanitizer. The stringency of government policies was measured with the help of information about restrictions on movement, school and business closures and so on together with information about the strictness of health containment policies enacted during this period. This information was used to produce a set of indicators that were then aggregated into an overall stringency index. The index has been used in a number of studies into the effect of policies on behaviour and the spread of the disease, and ranges between 0 and 100, with 100 indicating the most stringent response (Hale et al., 2021; also see the Supporting Information Appendix for details).^15^

**Figure 1:**
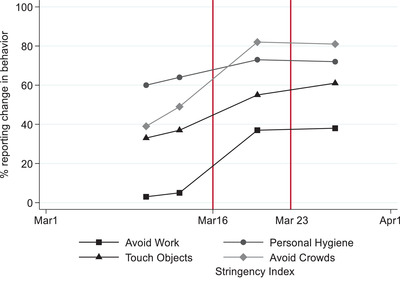
Government stringency and reports of changes in personal behaviour. [Colour figure can be viewed at wileyonlinelibrary.com] *Note*: Public opinion data were collected on 10, 13, 20 and 27 March, 2020. Stringency index data were collected daily.

Figure 1 plots the reported behaviours on a timeline (as connected lines), together with the level of government stringency described above (the shaded area). When we pinpoint the timing of government restrictions and reported behaviour in this way, we see that government restrictiveness increased significantly after 16 March and so did reports in hygiene and public health behaviours across the board, with social distancing seeing the biggest jump. Clearly, reports of changes in behaviour were consistent with the change in government policies.

While these data illustrate the plausibility of the argument, they have shortcomings, including of course that they capture reports of citizen behaviour rather than measures of actual behaviour. As a next step, I therefore sought to confirm reports of changes in behaviour with data on changes in people's actual behaviour. To do so, I tracked people's actual movements with the help of Google's COVID‐19 mobility reports in various categories. These data track individuals by location on a daily basis and measure the percentage change in the frequency of visits to and time spent in places like grocery stores and parks, transit, work and home, for instance, within a geographic area and relative to a pre‐COVID‐19 baseline (for further details, please see the Supporting Information Appendix ).^16^

**Figure 2:**
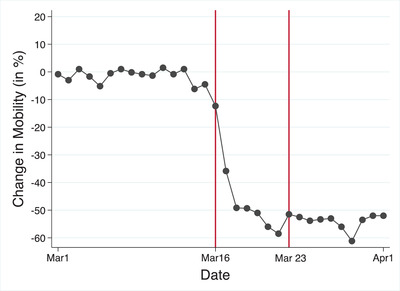
Changes in mobility in France. [Colour figure can be viewed at wileyonlinelibrary.com] *Note*: Google Mobility data were collected daily. Each dot represents a day's difference in mobility relative to the pre‐pandemic benchmark.

Figure 2 plots the changes in the movements of the French public between 1 March and 1 April, 2020. Keeping in mind that each dot represents one day, it reveals that, on the day prior to the Macron speech, people's movement had declined by 4.5 per cent relative to the pre‐pandemic baseline. This rose to −12 per cent on the 16th, followed by a precipitous decline to −36 per cent on the 17th and then to −50 per cent and below, after which it stayed at an extremely low level for the remainder of the month.

Overall, these patterns suggest a link between the actions of public authorities and people's private behaviours – specifically, that authorities’ actions reflected the public health threat and that citizens’ reported and actual behaviours mirrored government demands. The data also support my expectations about a clear and sudden break in behaviour following the speech by President Macron. In addition, they confirm that people's reports of changes in their behaviour were consistent with measures of changes in actual behaviour. Thus, taken together, these data provide prima facie evidence that French responded to the Covid 19 threat, that 16 March was an important juncture in the evolution of the pandemic in France, and that reports of past behaviour are highly likely to reflect actual changes in behaviour.

At the same time, they leave several questions unanswered: For example, do these patterns indicate that these changes in behaviour reflect the public's growing sensitivity to government demands and can we identify potential, specific government actions that may have driven these changes? In addition, how do citizens make sense of instructions from public authorities?

### Individual‐level analyses

**Figure 3:**
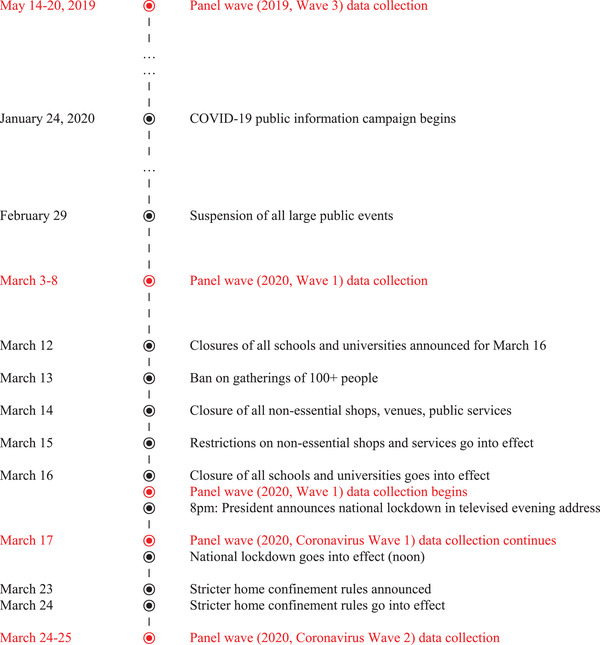
Timeline of French health and social distance measures and survey data collection [Colour figure can be viewed at wileyonlinelibrary.com]

Below, I address these questions further with the help of evidence collected via individual‐level panel surveys collected as part of the French National Election Study (Enquête Électorale Française – ENEF). Conducted since November 2015, these surveys are based on a panel of nationally representative sample of adults (Brouard, 2020). Fortunately, the timeline of government actions and data collection during March 2020 permits an examination of behaviours before and after key milestones in the government's pandemic response (Figure 3). In particular, surveys collected on 16–17 March and 24–25 March allow us to examine responses before and after the ‘we are at war’ speech by President Macron, as well as before and after the lockdown was formally enforced (in the time between the 16–17 March and 24–25 March survey waves).

To use experimental language, the surveys provide us with two treatments: the 16 March speech by Macron is one ‘treatment’ of the respondents, as one half of the 16–17 March survey wave was coincidentally interviewed before and the other after the speech. The subsequent lockdown constituted the second treatment, captured by a survey of all 16–17 March respondents who were re‐interviewed on 24–25 March. This means that the timeline of events produced one ‘between‐participants’ factor reflecting the Macron intervention (pre/post) and one ‘within‐participants’ factor – the lockdown (pre/post).^17^

Although the signal sent by President Macron's speech in terms of timing and content could hardly have been clearer, the observational nature of the study means that there are potential challenges to internal validity. For example, the analysis follows the “intention to treat” principle since Macron's speech was not watched by everyone. Moreover, assignment to the pre‐ or post‐speech group was an accident of timing and therefore not fully random. It is also plausible that respondents surveyed before the speech or before the government began to enforce restrictions anticipated a lockdown, given that people had been aware of the threat from the virus for a number of weeks. However, if anything, this should produce high levels of reported compliance before Macron's speech and therefore should make it more, rather than less, difficult to identify effect for the two treatments.

### Measuring compliance reports

The dependent variable measure relies on questions that asked people to report their social distancing and hygiene behaviours (for details, see Brouard, 2020 and Brouard et al., 2020). Respondents were asked whether, on a scale from 0 (not at all) to 10 (completely), they had changed their behaviour:

Because of the coronavirus epidemic, in your daily behavior, would you say that …?

‐ You wash your hands more often and/or longer.
‐ You cough or sneeze into your elbow or a handkerchief.
‐ You stopped greeting by shaking hands or kissing.
‐ You keep a distance of one meter from other people outside your home.
‐ You have reduced your trips.
‐ You avoid crowded places (public transport, restaurants, sports training …).
‐ You have stopped meeting your friends.

Exploratory factor analyses showed that these items also loaded highly on a single factor, allowing us to combine them into one behavioural index (for a similar approach, see Brouard et al., 2020; for further details as well as robustness tests, please see the Supporting Information Appendix ).^18^ Because these are reports of past behaviour, responses may combine actual changes in behaviour and some element of social desirability response (Hansen et al., 2021).^19^

## Compliance before and after the lockdown

**Figure 4:**
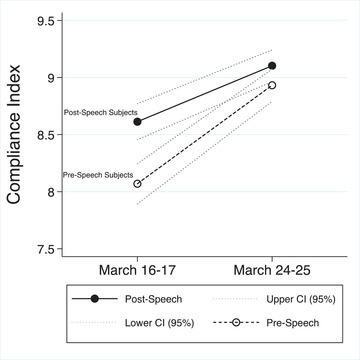
Compliance behaviours among respondents first interviewed before or after the 16 March speech by president macron. [Colour figure can be viewed at wileyonlinelibrary.com] *Note*: Pre‐speech subjects were interviewed before 8:00 PM on 16 March, while post‐speech subjects were initially interviewed after 8:00 PM on 16 and 17 March. All respondents were interviewed a once more on 24–25 March.

I used these measures to analyze behaviour in several steps. To see if there are differences between panel respondents’ reports of compliance before and after Macron's speech as well as before and after the formal lockdown, I take advantage of the fact that the timing of the 16–17 March survey wave meant that roughly half of the respondents were (quasi‐randomly) interviewed before the Macron speech and the other half after (there were only small differences in the composition of the two subsets of respondents; see Supporting Information Appendix A). This allowed us to examine the responses of subjects who were interviewed before or after the ‘treatment’ (i.e., respondents interviewed before and after the 8:00 PM televised address on 16 March), and to track their answers from one week to the next. Figure 4 reports the means across the two groups and measurements.

The graph reveals that the pattern I hypothesize is clearly reflected in the data: first, while levels of compliance were already high in the 16–17 March wave, there was a significant difference in compliance between respondents interviewed before and after the speech by President Macron (8.05 v. 8.61; difference in means: t = 5.30; p < 0.001). Moreover, the lockdown shifted everyone's compliance levels upward, regardless of whether they had been exposed to the speech. While those originally interviewed before the speech moved from 8.05 to 8.89 on the compliance scale the following week (t = 7.43; p < 0.001), respondents first interviewed after the speech moved from 8.61 to 9.08 post‐lockdown (t = 4.16; p < 0.001). However, results also showed that, by the second wave, the gap between those interviewed before and after the speech the week before had been reduced and was no longer statistically significant (t = 1.57; p = 0.116). Thus, the panel data suggest that citizens’ reported behaviour shifted following the speech by President Macron as well as the formal enforcement of lockdown rules.^20^

The patterns documented in Figure 4 provide evidence that the French government's persuasion efforts as well as the actual lockdown mattered for shifting people's understanding of the need to comply. First, the difference in reported compliance on 16 and 17 March between treated and untreated respondents is likely to result from the speech and thus the signalling by the French government regarding desirable behaviours. Results showed that the treatment shifted citizens’ compliance reports by half a point on the scale. Second, responses were high and then shifted even further upward during the week of the hard lockdown. Moreover, while they did so for both groups during the following week, the gap between them had largely disappeared by the time of the 24–25 March survey. I speculate that this means the message produced by the speech was overtaken by the formal lockdown, as well as non‐treated respondents learning about the speech.

## Individual‐level reports of compliant behaviour

To examine the individual‐level determinants of reported behaviours in more detail, I subsequently analyzed responses from the 16–17 March and 24–25 March panels with the help of multiple logistic regression estimations.

### Measures and estimation strategy

Given the high levels of compliance and associated skewness in the dependent variable reported above (with most responses clustering at the high end), I recoded the compliance index into a dummy variable (9 and above = 1; 0 otherwise).^21^ Regarding the independent variables, I measured presidential approval with the help of a question tapping people's evaluations of presidential performance, while co‐partisanship with the government is measured via attachment to “La République En Marche” (LREM), the centrist political party led by Emmanuel Macron that was unambiguously pulling the levers of state authority, having also won a clear majority of seats in the National Assembly in 2017 (Dageförde & Grossman, 2020).^22^

I measured attitudes toward the personal and societal risks posed by the virus with survey questions tapping into people's willingness to take personal health risks, as well as questions that asked respondents about the severity of the threat of the pandemic to France's public health and to the country's economy (see Supporting Information Appendix for question wording and coding details). Finally, I controlled for a set of individual‐level covariates that are likely to shape people's propensity to comply with government regulations or to engage in less risky health and social behaviours. These include respondents’ current health, demographic characteristics (age, sex, education, income, employment status and living with a partner) and extreme political ideology (for details, see the Supporting Information Appendix).^23^

I assessed the impact of my key independent variables in several ways. First, when possible, I included independent variables measured in panel waves prior to the dependent variable measures. For example, I used measures of long‐standing orientations like partisanship and left‐right self‐placement collected in a panel wave prior to the collection of compliance reports in the first March wave.^24^ Second, to ensure the robustness of the results, I estimated three specifications, including a simple logistic regression, a balanced model that pre‐treats the data using entropy balancing (Hainmueller, 2012) to reduce potential imbalance between respondents interviewed before or after the speech (see Supporting Information Appendix A for information about pre‐treatment sample balances), as well as a logistic regression with standard errors clustered at the level of region.^25^ Finally, because I expected the impact of the independent variables to vary as a function of exposure to messages and the presence of a formal lockdown, I estimated models for individuals surveyed before and after the speech by President Macron.^26^

### Estimating reports of compliance before and after the Macron speech

The results for models of voluntary compliance prior to the lockdown are reported in Table 1. They show, first, that political preferences indeed affected private behaviour, but these effects varied depending on whether respondents were surveyed before or after the 16 March speech. Specifically, I find that LREM partisans were more likely to report changes in their behaviour *prior* to 16 March, but there is no such effect after. In contrast, respondents who were satisfied with the president's performance were more likely to report engaging in compliance behaviour *after* the speech but not before.

**Table 1:** Determinants of reported compliance 16–17 March

	**Model 1**		**Model 2**		**Model 3**	
	Pre‐Speech	Post‐Speech	Pre‐Speech	Post‐Speech	Pre‐Speech	Post‐Speech
LREM Partisanship	0.623**	−0.004	0.615**	0.313	0.609**	0.313
	(0.375)	(0.396)	(0.326)	(0.387)	(0.345)	(0.379)
Presidential approval	0.020	0.134**	0.024	0.111**	0.017	0.111**
	(0.047)	(0.046)	(0.042)	(0.042)	(0.044)	(0.040)
Health risk acceptance	−0.110**	−0.175**	−0.098**	−0.152**	−0.086**	−0.152**
	(0.051)	(0.047)	(0.045)	(0.043)	(0.045)	(0.042)
Public health threat	0.942**	0.667**	1.054**	0.592**	1.057**	0.592**
	(0.228)	(0.173)	(0.195)	(0.153)	(0.183)	(0.141)
Economic threat	−0.251*	0.242	−0.333**	0.366**	−0.322**	0.366**
	(0.190)	(0.210)	(0.174)	(0.180)	(0.166)	(0.172)
Extreme left	0.033	−0.062	0.022	0.379	0.076	0.379
	(0.387)	(0.375)	(0.337)	(0.344)	(0.328)	(0.334)
Extreme right	−0.127	−0.440*	−0.084	−0.243	−0.067	−0.243
	(0.315)	(0.288)	(0.267)	(0.259)	(0.272)	(0.258)
Personal health	−0.073	0.081	−0.071	−0.028	−0.103	−0.028
	(0.155)	(0.136)	(0.134)	(0.117)	(0.131)	(0.117)
Female	0.981**	0.455**	0.957**	0.557**	0.993**	0.557**
	(0.232)	(0.222)	(0.217)	(0.201)	(0.224)	(0.201)
Age	0.022**	0.004	0.017**	0.006	0.016**	0.006
	(0.010)	(0.010)	(0.008)	(0.008)	(0.009)	(0.008)
Education	0.179	−0.119	0.066	−0.008	0.079	−0.008
	(0.142)	(0.144)	(0.126)	(0.122)	(0.123)	(0.117)
Income	−0.062	0.004	0.071	0.002	0.077	0.002
	(0.089)	(0.105)	(0.085)	(0.089)	(0.089)	(0.086)
Cohabits	0.397*	0.490**	0.182	0.480**	0.165	0.480**
	(0.276)	(0.271)	(0.241)	(0.231)	(0.252)	(0.233)
Employed	0.005	−0.187	0.010	−0.237	−0.002	−0.237
	(0.272)	(0.270)	(0.235)	(0.232)	(0.244)	(0.227)
Constant	−4.642**	−3.114**	−4.419**	−3.612**	−4.400**	−3.611**
	(1.113)	(1.084)	(1.002)	(0.967)	(1.037)	(0.970)
Observations	486	522	486	522	486	522
Groups					12	12
Chi‐square	62.239	74.413			69.514	69.754

Note: Standard errors in parentheses. Model 1: Logistic regression; Model 2: Balanced logistic regression; Model 3: Logistic regression with respondents clustered at region of residence.

^*^ *p* < 0.1,

^**^ *p* < 0.05 (one‐tailed tests of significance)

Calculations of the estimated substantive impact of these attitudes on voluntary compliance among typical respondents show that, LREM partisanship moved the predicted probabilities of high levels of compliance from 40 to 52 per cent prior to the speech. Presidential approval had similarly potent effects on responses about compliance after the speech. The likelihood that respondents who strongly disapproved of Macron also reported high levels of compliance were 48 per cent, while the likelihood among those who strongly approved was 70 per cent – a difference of 22 percentage points. Thus, the two political support measures have the expected but variable effects, with partisanship shaping compliance reports *before* and presidential approval *after* the speech.

Second, I find strong support for the hypothesis that people's risk aversion and perception of the public health risks influence whether people comply. Specifically, the willingness to take personal health risks is associated with significantly lower levels of compliance, especially after the speech. Moreover, those who identified the virus as a threat to public health were significantly more likely to report compliant behaviour both before and after the speech. Contrary to expectations, the impact of perceptions of the virus as a threat to economic health varied: Among those interviewed before the speech, viewing the virus as economic threat made them less likely to say they had complied with government recommendations, while it boosted compliance reports among respondents surveyed after the speech. This suggests that the Macron speech had the effect of highlighting the need to comply with the government's strategy in order to avert *both* a public health threat and negative consequences for France's economy.

Third, respondents’ own state of health did not significantly affect compliance, suggesting that appeals to protecting one's own health may not be especially effective. The effects for most of the other control variables are imprecisely estimated. For example, there is no evidence that ideological extremism, employment or education moved compliance, though living with a partner appears to have done so after the speech. The two individual‐level variables that mattered consistently were gender and age. Women are significantly more likely to comply before and after the speech (though the coefficient is smaller post‐speech), while age produced higher levels of compliance only before. The fact that women were significantly more likely to report following government guidelines is consistent with evidence that women are more likely to see COVID‐19 as a serious health problem, and also to agree and comply with public health measures (Galasso et al., 2020).^27^

Taken together, the estimations show that three factors mattered for reports of voluntary compliance prior to the formal lockdown: first, co‐partisanship with the government moved reported compliance prior to the Macron speech while presidential approval did so after; second, the perception of a public health threat increased compliance reports before as well as after the speech, while the perception of economic threat did so only after the speech; third, basic demographic characteristics matter relatively little, with the strongest effects for sex.

### Estimating compliance after the 23 March lockdown

**Figure 5:**
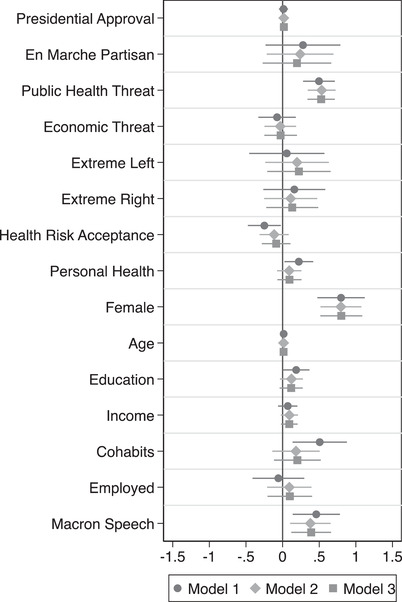
Determinants of voluntary compliance 24–25 March. *Note*: Unstandardized least squares coefficients; markers represent point estimates, while horizontal lines depict 95 per cent confidence intervals (one‐tailed).

To examine compliance following the lockdown announced on 23 March, I estimated a set of regression models using the surveys collected on 24 and 25 March. These are identical to those reported in Table 1 above (full results are shown in the Supporting Information Appendix), with the exception that I now include a dummy variable to control for the potential effect of the Macron speech the previous week, with those interviewed after the speech coded 1 (others 0). The results are shown in Figure 5 and reveal significant differences in the impact of the key variables on compliance just one week after the government moved to strictly enforce a tightened home confinement regime. Specifically, they reveal that partisanship and presidential approval no longer had statistically significant effects, while the impact of perceived public health threat decreased somewhat and perceptions of the virus as a threat to the French economy were no longer significant.

Interestingly, consistent with results reported above, the Macron speech appears to have had a lingering effect on survey responses, increasing the predicted probability of high levels of reported compliance after a full week of the lockdown by roughly 8 per cent. Taken together, these results indicate that, once the formal lockdown was in place, reported compliance was driven primarily by perceptions of the virus as a public health threat, differences between men and women and the lingering effects of the mobilization produced by the Macron speech, even if the exact psychological mechanism that drives these effects cannot be determined conclusively with the data at hand.^28^

To establish the robustness of the findings, I examined a variety of potential challenges to the inferences, relating to the data, measures and estimation methods. Specifically, I investigated whether the results were driven by specific compliance behaviours, whether political attitudes predicted placebo outcomes (unrelated private behaviours), the nature of partisanship in France and whether attachments to other parties played a role as well. These additional analyses are reported in the Supporting Information Appendix and consistently confirm the robustness of the findings reported above.

## Conclusion

‘We are at war, in a health war,’ French President Emmanuel Macron declared in a primetime speech to the French nation at 8:00 PM on 16 March, 2020. By the middle of March 2020, as it became clear that France was among the countries hit earliest and hardest by the global Covid‐19 pandemic, the French government had already implemented severe restrictions on travellers from abroad, systematic limits on public gatherings and a string of other restrictions on workplaces, schools and social interactions. As the President was about to announce new and extremely stringent home confinement rules, France found itself in a moment of acute crisis.

As we know now, France was hit hard but also was hardly exceptional: as the pandemic raced across the globe, governments everywhere tried to fight the highly contagious virus by asking citizens to comply with recommendations to change private behaviours as well as to acquiesce to severe limits on basic civil liberties. To make such policies effective, governments needed citizens to comply with rules and restrictions, ideally without being forcibly compelled. After all, when compliance is not uniform, the effectiveness of government is likely to be compromised. Democracies, in particular, rely on such cooperation – yet, many of the changes in behaviours needed to fight a pandemic take place out of sight of the state. Getting people to comply when no one is watching is easier when they have faith in the government and its ability to make credible commitments to ensure collective welfare. Because this sounds so plausible as to border on tautology, I sought to be more precise about the ways in which state authority and citizen attitudes combine to produce compliance with government demands.

Specifically, I posited that the exercise of public authority and individual‐level attitudes combine to shape people's willingness to protect themselves and others. Moreover, I argued that times of crisis are especially important moments to understand from a theoretical and empirical perspective because they challenge the ability of democratic states to mobilize their populations when threatened. While governments routinely seek to induce citizens to comply with a wide variety of government demands – from eating healthier foods to recycling more, to name just a couple of mundane examples – the micro‐foundations of everyday health and social behaviours during times of crisis are less well understood. In fact, acute crises provide the context for a particularly hard test for locating the drivers of acquiescence to state demands, given the high levels of uncertainty surrounding the effectiveness of government policies.

While the idea that risk‐related beliefs influence people's adherence to government demands during times of acute crisis may be relatively straightforward, the expectation that political attitudes affect people's compliance is not altogether obvious. After all, the behaviours governments have sought to shape are habitual decisions related to things like personal hygiene (e.g., washing hands, sneezing), rituals of social interactions (e.g., greeting others) or movement (e.g., mode of travel, distancing while talking a walk). Not only are these behaviours far removed from politics or the long arm of the state, but having one's private life, habits and decisions redirected by government regulations may not be the same as being encouraged to eat healthier foods or saving more for retirement, however. In fact, research on nudges and behavioural public policy has provided mixed results when it comes to the efficacy of short‐term signals to change behavioural intentions, given the strong element of habit.

I examined my hypotheses with data from France. Results show that governments indeed have the power to move behaviour via messages and enforced compliances, and that support for incumbents and perceptions of threat to collective health have the potential to shape adherence to the new rules. The findings therefore suggest that political preferences help us understand the connection between citizens and the state, and that support for incumbent governments generally and partisanship in particular can buttress people's commitment to basic civic obligations – the duty to protect one's fellow citizens.

These results are consistent with research about the effect of partisanship on ostensibly non‐political behaviour, including consumption and social behaviour, as well as nascent research on the partisan foundations of opinions about the Covid‐19 pandemic in the United States, for instance (Cornelsen & Miloucheva, 2020; Gadarian et al., 2021 findings). Implicitly and explicitly, partisan divisions in compliance and attitudes about the pandemic in the United States have been chalked up to a very polarized political environment. While results suggest that partisanship has the potential to move private behaviour even when there is elite consensus and limited polarization in the electorate, I also found that partisanship mattered only before President Macron spoke to the French nation on 16 March – that is only before the state began to escalate its crisis response. In contrast, views of presidential performance in office mattered only after his highly publicized intervention using the symbols of state power.

Because partisanship is an ongoing relationship with political actors as part of someone's enduring political identity, it is a heuristic people use when monitoring information and signals from political authorities on a routine basis and absent a crisis signal. However, once the President speech raised the emergency alarm, it was belief in the executor of political authority that served as a heuristic for incentivizing behaviour that would not otherwise happen and for providing a focal point for coordinating behaviour that might happen but in an inefficient or haphazard fashion if left decentralized. Interestingly, the finding that partisanship disappeared as a significant influence on reported compliance stands in stark contrast to the US case where partisanship has been such a consistent driver of Covid‐19 related attitudes and behaviours. I speculate that the less polarized political environment of the French case facilitated the disappearance of partisanship as a main driver once the crisis intensified.

There are likely to be important scope conditions to the findings reported here. For example, the French case may be unique in that the martial tone and the content of President Macron's speech highlighted the need to mobilize the nation in support of a state of sanitary emergency, cueing citizens to perceive the crisis as a health crisis, and putting scientists and doctors’ recommendations above any others to guide policy decisions. Moreover, the French government was among the more heavy‐handed in its strict enforcement of the lockdown restrictions anywhere in Europe.

Furthermore, the combined timing of the Macron speech and the survey measurements meant that the ‘treatment’ effects were especially strong. In this way, this study focuses on an extreme moment in an already extraordinary period of time. And while the lingering effects of the speech on individuals’ levels of compliance in the subsequent survey wave is clear, there is no unequivocal evidence on the precise ways in which Macron's address contributed to this effect – for example, whether this was because of the nature of the speech, its messaging, because of the penalties for violating the rules, a boost in government approval or simply a sense of citizen duty. Related to this, I would speculate that the impact of some of the independent variables on compliance behaviours may have shifted as the pandemic progressed; for example, while public health concerns may have been uppermost on citizens’ minds in the middle of March, the government policies’ effects on the economy, schools or civic life may have become more prominent into the summer.

In the end, the findings suggest that the state has multiple levers to activate compliance, ranging from persuasion via messages by political leaders and public health officials to coercion via police patrolling the streets. At the same time, it is clear that not everyone is equally receptive to the messages or to following orders. Thus, in combination, in times of crisis, the long arm of the state is really more of a hydra that has the capacity to embrace citizens in multiple ways, but whose efficacy at engendering compliance depends on citizens’ motivations to listen in the first place.

## Supporting information

Table A.1. Variables and Coding for Individual‐Level Survey Data
Figure A.1. Box Plots of Compliance Behaviors
Table A.2. Descriptive Statistics, Individual‐Level Data
Table A.3. Summary Statistics Before Entropy Balancing
Table B.1a. Determinants of Compliance Before President Macron's Speech
Table B.1b. Determinants of Compliance After President Macron's Speech
Table B.2. Determinants of Private Behaviors March 24‐25
Table B.3. Individual‐Level Stability of Partisan Attachments in France
Table B.4. Individual‐Level Stability of Partisan Attachments in France
Figure B.1. Effects of Partisanship on Compliance Behaviors

Supplementary information

## Notes

1 During normal times, this cooperation may include anything from sending children to school or serving in the military to driving on the correct side of the road (Levi, 1988, 1997; Lieberman, 2009; Tyler, 1990; Tyler & Huo, 2002).

2 Researchers have studied the relationship between citizens and the state using different empirical and theoretical approaches (see Gordon & Huber, 2019 for a review). Based on a variety of normative and empirical understandings, scholarship across the social sciences has produced qualitative and quantitative evidence of the sources and consequences of consent for democratic as well as non‐democratic societies and has done so with the help of surveys, experiments, participant observation and archival work.

3 Although taxation is perhaps the most common form of material sacrifice the state demands of citizens, it clearly is only one of many, including military conscription or jury duty, for example. States have developed mechanisms to make such sacrifices more automatic and more easily enforceable, and thus less voluntary. For instance, in many countries, income taxes are withheld at the source (by an employer), making evasion and non‐compliance more difficult though not impossible. Thus, even in democracies, what look like voluntary contributions often occur in the shadow of coercion.

4 I bracket the question of social (or horizontal) trust as a precursor to engaging in collectively beneficial behaviour, as I focus on the relationship between citizens and the state and because there is a large independent literature on this topic (e.g., Norris et al., 2008), including a growing stream of research during the Covid‐19 pandemic (Borgonovi & Andrieu, 2020; Oksanen et al., 2020).

5 Airport screening was mandated as of 23 January; the public health campaign commenced on 24 January, 2 days before the first cases became public; and quarantines on persons arriving from high‐risk regions were instituted on 31 January.

6 There were exceptions relating to professional activity, buying essential goods, for health or family reasons or brief movements for individual physical exercise.

7 Macron's speech combined an announcement of new rules and restrictions, a call to arms for the French people to show solidarity in order to defeat the virus collectively, as well as a set of specific benefits designed to protect French businesses and individuals.

8
https://franceintheus.org/spip.php?article9659

9 President Macron's 16 March speech was seen by an estimated 35 million viewers, the largest TV audience in French history. http://www.leparisien.fr/culture‐loisirs/coronavirus‐35‐millions‐de‐francais‐devant‐l‐allocution‐de‐macron‐un‐record‐d‐audience‐absolu‐17‐03‐2020‐8281773.php

10 This represents the percentage of respondents who said they were ‘very’ or ‘somewhat’ scared to contract the virus: https://yougov.co.uk/topics/international/articles‐reports/2020/03/17/fear_catching‐Covid‐19 .

11 Narrowly defined, nudging is not about information processing – that is, the effect of political biases and heuristics. Rather, it is about changing people's choice environment “so that when people follow their instincts, using familiar mental shortcuts, the most prominent option available to the policy target will be one that is likely to promote their own welfare, and that of society more widely” (Moseley, 2020).

12 Along these lines, data collected in the United States suggest that reactions to government's handling of the COVID‐19 pandemic have been highly partisan – in particular, people's perceptions of the severity of the crisis, whether government was doing a good job and whether they adjusted their personal behaviour, such as engaging in social distancing, for instance (Allcott et al., 2020; Gadarian et al., 2021) – though the evidence outside the United States has been more mixed. For example, Canadian studies have reported only modest or no evidence of partisan divisions (Merkley et al., 2020; Pickup et al., 2020) and a British study showing no distinct partisan effects on people's willingness to engage in protective measures (Anderson & Hobolt, 2021).

13 There is some evidence that a related construct – political trust – is correlated with compliance at the aggregate level of regions (Bargain and Aminjonov 2020) as well as the individual level in the United States (Robinson et al. 2020).

14 Respondents were asked: ‘Thinking about the last 7 days& how often have you taken the following measures to protect yourself or others from coronavirus (COVID‐19)? As a reminder, please exclude any measures that you have already taken for reasons other than coronavirus’:‐ *avoided touching objects in public (e.g. elevator buttons or doors),‐ avoided crowded areas,‐ avoided working outside your home,‐ washed hands with soap and water; used hand sanitizer*.For further details, please see the supplementary appendix.

15 See also https://www.bsg.ox.ac.uk/research/research‐projects/coronavirus‐government‐response‐tracker and https://www.bsg.ox.ac.uk/sites/default/files/2020‐05/BSG‐WP‐2020‐032‐v6.0.pdf.

16 Further information about these data can also be found here: https://www.google.com/covid19/mobility/ and here https://www.gstatic.com/covid19/mobility/2020‐06‐19_FR_Mobility_Report_en.pdf.

17 The within‐respondent analysis is restricted to individuals who answered both waves so that the N is the same for each wave.

18 I conducted exploratory factor analyses for behaviours in the two waves separately. In both waves, only one factor achieved an Eigenvalue greater than 1 (wave 1: 4.32; wave 2: 3.13). The combined scale had very high reliability (Cronbach's alpha was 0.89 and 0.77 for reported behaviours in waves 1 and 2, respectively). (Please see the Supporting Information Appendix for more details)

19 Reporting compliance in line with messages from public authorities stems from a variety of sources, all of which result in respondents reporting appropriate behaviours when asked about hygiene and social distancing behaviours as defined by government messages. This includes the effect of information, social desirability bias or so‐called virtue‐signalling. Existing evidence of an over‐reporting response bias is mixed. While Daoust et al. (2021) report bias ranging from 8 to 11 per cent in the Canadian case and other studies diagnose differences in reported non‐compliance rates cross‐nationally (Becher et al., 2020). Munzert and Selb (2020) and Larsen et al. (2020) find little to no evidence of bias in Germany and Denmark, respectively. Some scholars (Becher et al.m 2020; Daoust et al.m 2020; Munzert & Selb, 2020) have sought to diagnose and remedy the potential for under‐reporting of non‐compliance, if it indeed exists, with so‐called list experiments, face‐saving questions or different question formats. To the extent that it is a concern, it should primarily affect mean responses and intercepts, rather than slope estimates.

20 Higher levels of compliance post‐lockdown may partially be engendered by a willingness to comply for the sake of compliance in reaction to signals sent by the government, as I hypothesize, but may of course also simply be a function of not wanting to pay fines.

21 The distribution is shown in Supporting Information Appendix A.

22 The zero‐order correlation between approval and LREM partisanship was 0.43 in the 16–17 March wave and 0.41 in the 23–24 March wave, suggesting that partisanship explained 17–18 per cent of the variation in approval responses.

23 I also experimented with additional variables, including community size, religious affiliation, happiness and social trust. None of these achieved statistical significance and were not investigated further.

24 In the case of partisanship, I was able to rely on a survey conducted 3–7 March. While the survey conducted 3–8 March did not include questions about presidential support, the 16 March wave did. I therefore used approval collected as part of the latter panel. The zero‐order correlation between approval measured in May 2019 and March 2020 was 0.87, suggesting a high degree of stability. Tests showed that approval measured in May 2019 had similar, but slightly weaker effects on the dependent variables.

25 The incidence of COVID‐19 was uneven across French administrative regions, with the region centred on Paris (Île de France) and the Northeast of the country reporting significant greater outbreaks. I estimated a series of models using regional fixed effects as a robustness checks; these results were essentially identical.

26 There are two ways to deal with qualitative independent variables that represent two populations and that are hypothesized to have an interactive effect with another independent variable on the dependent variable of choice: stratifying the sample into two subsamples or specifying the behavioural differences within the overall model with the help of interaction terms. The use of interaction terms assumes that the other behavioural relationships are the same across observations; that is, that the other independent variables have essentially identical effects in both populations. Because I have reason to assume that the other behavioural relationships were dissimilar across observations, I employed separate regressions instead of interactive terms. The different effects for economic threat, sex or health risk acceptance demonstrate that this concern was justified.

27 Importantly, Galasso et al. (2020 find that these results are not driven by social desirability bias, and they dovetail with evidence in infection and disease patterns, with women consistently less affected by the virus than men (Wenham et al., 2020).

28 One possibility is that lockdowns increase trust in government and support for the incumbent (Bol et al., 2020; de Vries et al., 2021). One implication might be that enhanced political trust produced provides the mechanism explaining the relationship between the enforcement of restrictions and compliance.
