# Supplementary material for: Citizens and the state during crisis: Public authority, private behaviour and the Covid‐19 pandemic in France
Source: Eur J Polit Res. 2022 Mar 26:10.1111/1475-6765.12524. Online ahead of print. doi: 10.1111/1475-6765.12524 (PMC9111144; doi:10.1111/1475-6765.12524)
Supplement: Supplementary file 1 — Table A.1. Variables and Coding for Individual‐Level Survey Data Figure A.1. Box Plots of Compliance Behaviors Table A.2. Descriptive Statistics, Individual‐Level Data Table A.3. Summary Statistics Before Entropy Balancing Table B.1a. Determinants of Compliance Before President Macron's Speech Table B.1b. Determinants of Compliance After President Macron's Speech Table B.2. Determinants of Private Behaviors March 24‐25 Table B.3. Individual‐Level Stability of Partisan Attachments in France Table B.4. Individual‐Level Stability of Partisan Attachments in France Figure B.1. Effects of Partisanship on Compliance Behaviors [file EJPR-9999-0-s002.pdf]

Anderson, Christopher J. 2022. "Citizens and the State During Crisis: Public Authority, Private Behavior, and the Covid-19 Pandemic in France" ***European Journal of Political Research.***

Supplemental Appendix

Appendix A. Survey Data and Government Policies

This section provides additional information about the data sources, variable measures, and descriptive statistics.

Aggregate Surveys

The aggregate survey data were collected by YouGov in four separate surveys on March 10, 13, 20, and 27 of 2020. The specific questions asked respondents: "Thinking about the last 7 days... how often have you taken the following measures to protect yourself or others from coronavirus (COVID-19)? As a reminder, please exclude any measures that you have already taken for reasons other than coronavirus"

- Avoided touching objects in public (e.g. elevator buttons or doors)
- Avoided crowded areas
- Avoided working outside your home
- Washed hands with soap and water; used hand sanitizer

For further details, see: https://yougov.co.uk/topics/international/articles-reports/2020/03/17/YouGov-international-COVID-19-tracker

Government Policies

The stringency of government policies measure was collected by researchers at the University of Oxford who collected and coded publicly available information on 17 indicators of government responses. Eight of the policy indicators record information on

containment and closure policies, such as school closures and restrictions in movement. Four of the indicators record economic policies, such as income support to citizens or provision of foreign aid. Five of the indicators record health system policies such as the COVID-19 testing regime or emergency investments into healthcare. The data from the 17 indicators are aggregated into a set of four common indices, reporting a number between 1 and 100 to create an overall government response index as well as specific indices measuring responses in the areas of containment and health index; economic support, and stringency (which records the strictness of 'lockdown style' policies that primarily restrict people's behavior). The analysis utilizes the overall stringency index, which combines information about restrictions on movement, school and business closures, and so on together with information about the strictness of health containment policies. For further details on coding, see https://www.bsg.ox.ac.uk/research/research-projects/coronavirus-government-response-tracker. (also see https://www.bsg.ox.ac.uk/sites/default/files/2020-05/BSG-WP-2020-032-v6.0.pdf for further details on the index).

Individual-Level Data and Measures

To examine individual-level attitudes and behaviors, the analysis relies on panel surveys collected as part of the French National Election Study (Enquête Électorale Française- ENEF). These surveys are based on a panel of nationally representative samples of adults. I used data from three panel waves collected in March 2020, supplemented with data on respondent characteristic recorded in prior years (Brouard 2020).

The data are available for download from:

https://data.sciencespo.fr/dataset.xhtml?persistentId=doi:10.21410/7E4/EATFBW

The variables and coding are shown in the table below.

Table A.1. Variables and Coding for Individual-Level Survey Data

Compliance Behaviors	"Because of the coronavirus epidemic, in your daily behavior, would you say that ...?"<br>- You wash your hands more often and / or longer<br>- You cough or sneeze into your elbow or a handkerchief<br>- You stopped greeting by shaking hands or kissing<br>- You keep a distance of one meter from other people outside your home<br>- You have reduced your trips<br>- You avoid crowded places (public transport, restaurants, sports training ...)<br>- You have stopped meeting your friends<br>Respondents were asked to indicate agreement or disagreement on a 0 to 10 scale, with "0" meaning "no, not at all" and "10" meaning "yes, absolutely".
Presidential Approval	Based on question asking respondents to rate how satisfied they were with the performance of the President on a scale from 0 to 10, with 0 indicating "absolutely not satisfied" and 10 indicating "absolutely satisfied".
LREM Partisanship	Based on a series of questions asking respondents to indicate whether there is a party they feel close to or less distant from, and then to indicate which party that is.
Public Health Threat	Based on a question asking respondents: "Would you say that the consequences of the coronavirus epidemic on health in France today are ...?<br>1. Very serious<br>2. Rather serious<br>3. Moderately severe<br>4. Not very serious<br>5. Not at all serious<br>This variable was reverse-coded, with 5 indicating "very serious".
Economic Threat	Based on a question asking respondents: "Would you say that the consequences of the coronavirus epidemic on the economy in France today are ...?<br>1. Very serious<br>2. Rather serious<br>3. Moderately severe<br>4. Not very serious<br>5. Not at all serious<br>This variable was reverse-coded, with 5 indicating "very serious".
Extreme Left	Based on a question asking respondents: "On a scale of 0 to

	10, where 0 corresponds to the left and 10 corresponds to the right, where would you say you are?" Respondents were recoded so that 0-2 were coded as extreme left.
Extreme Right	Based on a question asking respondents: "On a scale of 0 to 10, where 0 corresponds to the left and 10 corresponds to the right, where would you say you are?" Respondents were recoded so that 8-10 were coded as extreme right.
Health Risk Acceptance	Based on survey question that asked: "Is it easy or difficult for you to accept or take risks that … affect your health"? Respondents could indicate on a scale from 1 to 10, 1 indicating "very difficult" and 10 "very easy". This variable was recoded to indicate "difficult" (1), "neither easy, nor difficult" (2), and "difficult" (3).
Personal Health	Based on survey question that asked: "Generally speaking, you would say that your health is ..."<br>1. Very good<br>2. Good<br>3. Fairly good<br>4. Bad<br>5. Very bad<br>This variable was reverse-coded, with 5 indicating "very good".
Sex	Female (1), male (0)
Age	Actual age (in years)
Education	Based on highest level of formal education completed, ranging from low to high.<br>Aucun diplôme, CEP (1)<br>BEPC, CAP, BEP (2)<br>BAC (3)<br>Diplôme Supérieur (4)
Income	Monthly income (in euros)
Cohabits	Lives with partner (1), lives alone or without a partner (0).
Employed	In full-time or part-time employment (1), otherwise (0).

Measuring Compliance Behaviors

Because the measure of compliance is based on people's reported behavior rather than their observed behavior, validity and reliability concerns cannot be completely ruled out. However, this shortcoming is mitigated in several ways. First, asking about specific health and social behaviors is simple and easy to understand. Second, the survey asks respondents about recent behavior during moments of acute crisis; as such, the context is vivid and the question should not require much thought. Third, given that there is widespread public evidence that the French public did indeed comply with government demands, high levels of reported compliance provide meaningful face validity that the question indeed measures compliance.

Factor analyses showed that these items also loaded highly on a single factor. Specifically, I conducted factor analyses for these behaviors in the two waves separately. In both waves, only one factor achieved an Eigenvalue greater than 1 (wave 1: 4.32; wave 2: 3.13). I combined these items into a scale that averaged responses across the items. The combined scale (also ranging from 0 to 10) had very high reliability (Cronbach's alpha was 0.89 and 0.77 for reported behaviors in the March 16 and March 24 waves, respectively).

Figure A.1. Box Plots of Compliance Behaviors

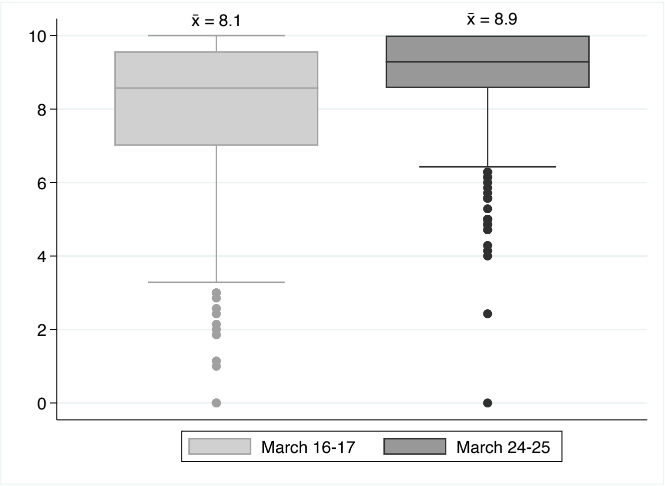

Table A.2. Descriptive Statistics, Individual-Level Data

Variable	Mean	SD	Min	Median	Max
Compliance Behavior (Mar 16-17)	8.34	1.89	0	9	10
Compliance Behavior (Mar 24-25)	8.95	1.42	0	9.4	10
Presidential Approval (Mar 16-17)	4.48	2.9	0	5	10
Presidential Approval (Mar 24-25)	4.21	2.9	0	5	10
LREM Partisanship	0.11	.32	0	0	1
Public Health Threat	4.32	.79	1	4	5
Economic Threat	4.53	.69	1	5	5
Extreme Left	0.12	.32	0	0	1
Extreme Right	0.18	.39	0	0	1
Health Risk Acceptance	1.68	.69	1	2	3
Personal Health	3.66	.85	1	4	5
Sex	0.53	.50	0	1	1
Age	54.75	14.77	20	57	92
Education	3.16	.93	1	3	4
Income	3.37	1.40	1	4	6
Cohabits	0.64	.48	0	1	1
Employed	0.44	.50	0	0	1

Entropy Balancing

In addition to using the population weighted sample for the multiple regression models, the estimations also include a so-called balanced specification. This procedure uses entropy balancing (Hainmueller 2012) to reduce any imbalance between respondents interviewed before or after the speech by President Macron. Table A.3 shows the descriptive statistics of for the outcome and control variables used in the analysis. It also separates respondents by whether they were surveyed before or after 8pm on March 16 and provides information about the sample's potential imbalance before and after the speech. This imbalance appears small for most variables (including age, education, income, employment, and living with a partner), and slightly larger for sex, which may bias estimates if not corrected. I therefore also use a specification that weights observations through entropy balancing (so called Balance specification), which helps to increase internal validity by weighting the distribution of covariates among non-treated respondents to make it mimic the first and second moment of the equivalent distribution among treated respondents.

Table A.3. Summary Statistics Before Entropy Balancing

	Before Speech				After Speech			
**Variable**	Min	Max	Mean	SD	Min	Max	Mean	SD
**Compliance Behavior**								
Compliance March 16-17	0	10	8.05	1.958	0	10	8.61	1.784
Compliance March 24-25	0	10	8.89	1.408	2.86	10	9.08	1.339
**Attitudinal Variables**								
Presidential approval	0	10	4.25	2.886	0	10	4.69	2.799
LREM partisan	0	1	0.14	0.347	0	1	0.10	0.300
Public health threat	1	5	4.33	0.795	1	5	4.31	0.788
Economic threat	1	5	4.48	0.766	2	5	4.58	0.608
Extreme left	0	1	0.14	0.342	0	1	0.10	0.305
Extreme right	0	1	0.21	0.408	0	1	0.18	0.388
Personal health risk	1	3	1.70	0.704	1	3	1.66	0.685
Subjective health	1	5	3.66	0.832	1	5	3.66	0.865
**Socio-Demographics**								
Sex	0	1	0.52	0.500	0	1	0.56	0.496
Age	20	92	55.06	14.665	20	92	53.98	15.230
Education	1	4	3.18	0.925	1	4	3.14	0.939
Monthly income	1	6	3.41	1.443	1	6	3.39	1.356
Living with partner	0	1	0.65	0.479	0	1	0.67	0.472
Employed	0	1	0.45	0.498	0	1	0.43	0.496
Observations			488					522

Appendix B. Supplemental Analyses and Robustness Tests

To establish the robustness of the findings, this section examines potential challenges to the inferences. These relate to the data, measures, and estimation methods.

Estimations of Individual Behavioral Items

To confirm that the effects of political and policy attitudes are consistent across the range of behaviors that make up the compliance index, I estimated a set of models for each compliant behavior separately. These are shown in Table B.1. Even though the impact varies, the conclusions reported in the paper are consistently supported across the different behaviors.

Table B.1a. Determinants of Compliance Before President Macron's Speech

	Wash Hands	Sneeze	Greet	Social Distance	Travel	Attend Gatherings	Meet Friends
Presidential	0.004	-0.016	0.009	-0.043	-0.010	0.013	0.008
Approval	(0.043)	(0.040)	(0.045)	(0.041)	(0.041)	(0.042)	(0.041)
LREM	0.764**	0.658**	0.497*	0.588**	0.678**	0.847**	0.338
Partisanship	(0.355)	(0.340)	(0.374)	(0.332)	(0.347)	(0.369)	(0.319)
Public Health	0.807**	0.384**	0.883**	0.649**	0.931**	0.776**	0.764**
Threat	(0.160)	(0.142)	(0.163)	(0.162)	(0.167)	(0.164)	(0.186)
Economic Threat	-0.043	0.057	-0.076	0.012	-0.210*	-0.049	-0.091
	(0.159)	(0.145)	(0.162)	(0.165)	(0.159)	(0.156)	(0.167)
Extreme Left	0.718**	0.151	0.283	0.709**	-0.138	0.307	0.016
	(0.362)	(0.325)	(0.375)	(0.324)	(0.319)	(0.329)	(0.324)
Extreme Right	0.163	-0.109	-0.374*	0.175	-0.176	-0.178	-0.091
	(0.283)	(0.252)	(0.279)	(0.253)	(0.267)	(0.275)	(0.252)
Health Risk	-0.185	-0.073	-0.164	-0.208*	-0.346**	-0.406**	-0.010
Acceptance	(0.152)	(0.144)	(0.168)	(0.151)	(0.152)	(0.155)	(0.150)
Personal Health	-0.069	-0.116	-0.136	0.098	0.000	-0.131	-0.122
	(0.129)	(0.122)	(0.135)	(0.129)	(0.128)	(0.131)	(0.122)
Sex	0.719**	0.830**	0.706**	0.450**	0.799**	0.699**	0.597**
	(0.219)	(0.209)	(0.232)	(0.208)	(0.211)	(0.219)	(0.203)
Age	0.032**	0.008	0.026**	0.032**	0.020**	0.018**	0.012*
	(0.008)	(0.008)	(0.009)	(0.008)	(0.008)	(0.008)	(0.008)
Education	0.195*	0.060	0.416**	0.137	0.081	0.020	0.152
	(0.122)	(0.113)	(0.126)	(0.117)	(0.120)	(0.123)	(0.120)
Income	-0.048	-0.023	0.116	-0.056	-0.026	0.042	-0.022
	(0.082)	(0.081)	(0.095)	(0.083)	(0.085)	(0.083)	(0.084)
Cohabits	0.260	0.149	-0.338	0.231	0.154	0.075	0.065
	(0.246)	(0.233)	(0.279)	(0.242)	(0.248)	(0.248)	(0.241)
Employed	-0.034	-0.139	0.320*	0.192	0.164	-0.239	-0.023
	(0.227)	(0.221)	(0.243)	(0.216)	(0.227)	(0.228)	(0.218)
Constant	-5.389**	-1.987**	-5.261**	-5.296**	-4.254**	-3.146**	-4.195**
	(1.102)	(0.965)	(1.130)	(1.107)	(1.114)	(1.063)	(1.092)
Observations	486	486	486	486	486	486	486
Chi-square	82.187	41.089	86.247	58.745	74.462	70.659	42.446

Note: Results are logistic regression estimates, with responses weighted by sampling weights. Standard errors in parentheses. * $p < 0.1$, ** $p < 0.05$ (one-tailed)

Table B.1b. Determinants of Compliance After President Macron's Speech

	(1)<br>Wash<br>Hands	(2)<br>Sneeze	(3)<br>Greet	(4)<br>Social<br>Distance	(5)<br>Travel	(6)<br>Attend<br>Gatherings	(7)<br>Meet<br>Friends
Presidential	0.030	0.062*	0.064*	0.018	0.074**	0.059*	0.092**
Approval	(0.039)	(0.039)	(0.046)	(0.040)	(0.041)	(0.041)	(0.040)
LREM	-0.066	-0.026	-0.587*	-0.022	-0.113	0.020	-0.269
Partisanship	(0.359)	(0.373)	(0.446)	(0.362)	(0.370)	(0.441)	(0.391)
Public Health	0.575**	0.682**	0.596**	0.480**	0.584**	0.335**	0.614**
Threat	(0.140)	(0.130)	(0.148)	(0.142)	(0.141)	(0.136)	(0.141)
Economic Threat	0.264*	-0.065	0.300**	0.287**	0.156	0.459**	0.414**
	(0.169)	(0.165)	(0.177)	(0.164)	(0.170)	(0.171)	(0.172)
Extreme Left	-0.386	-0.113	-0.028	0.281	-0.109	0.394	0.780**
	(0.310)	(0.311)	(0.412)	(0.327)	(0.328)	(0.382)	(0.361)
Extreme Right	-0.204	-0.044	-0.468*	-0.072	-0.289	-0.179	0.121
	(0.258)	(0.259)	(0.309)	(0.267)	(0.262)	(0.267)	(0.259)
Health Risk	-0.148	-0.307**	-0.409**	-0.165	-0.380**	-0.478**	-0.358**
Acceptance	(0.145)	(0.145)	(0.178)	(0.145)	(0.147)	(0.158)	(0.154)
Personal Health	0.048	0.136	0.072	-0.061	-0.028	-0.094	-0.033
	(0.115)	(0.121)	(0.142)	(0.116)	(0.116)	(0.124)	(0.118)
Sex	0.699**	0.483**	0.589**	0.259*	0.636**	0.290*	0.634**
	(0.199)	(0.201)	(0.244)	(0.199)	(0.200)	(0.219)	(0.202)
Age	0.016**	0.008	0.026**	0.023**	0.008	0.006	0.005
	(0.008)	(0.008)	(0.010)	(0.008)	(0.008)	(0.008)	(0.008)
Education	-0.060	0.112	0.198*	0.088	-0.065	-0.077	0.088
	(0.121)	(0.118)	(0.146)	(0.121)	(0.119)	(0.131)	(0.128)
Income	0.065	-0.064	0.124	0.065	0.107	0.094	0.012
	(0.091)	(0.090)	(0.117)	(0.088)	(0.089)	(0.097)	(0.094)
Cohabits	0.202	0.142	0.199	0.305*	-0.007	0.255	0.253
	(0.230)	(0.231)	(0.279)	(0.225)	(0.230)	(0.237)	(0.245)
Employed	0.278	0.148	-0.088	-0.042	-0.050	-0.149	-0.067
	(0.217)	(0.217)	(0.257)	(0.214)	(0.218)	(0.231)	(0.223)
Constant	-4.565**	-3.087**	-4.902**	-4.358**	-3.019**	-2.226**	-4.698**
	(1.150)	(1.135)	(1.313)	(1.137)	(1.113)	(1.150)	(1.197)
Observations	522	522	522	522	522	522	522
Chi-squared	57.183	48.068	59.838	50.573	53.595	45.686	62.411

Note: Results are logistic regression estimates, with responses weighted by sampling weights. Standard errors in parentheses. * $p < 0.1$, ** $p < 0.05$ (one-tailed)

Placebo Tests

I also undertook several placebo tests to establish that the relationship between individual attitudes and characteristics and subsequent reports of voluntary compliance is not simply part of a broader syndrome of behavioral adjustments or survey responses during times of crisis. If exposure to the pandemic and government announcements change reports of behaviors not explicitly related to the restrictions enacted by governments, it would be misleading to interpret the findings above as the effect of government policies on voluntary compliance.

To examine this possibility, I estimated identical models (in terms of explanatory variables) for placebo outcomes related to changes in other kinds of behaviors in the wake of the threat posed by the pandemic. These include stocking up on provisions, drinking more alcohol, and cleaning the home more frequently than before the crisis. Results reported in Table B.2. show that the political variables had few and inconsistent effects – while presidential approval reduces the odds of cleaning the home, LREM partisanship reduces the propensity to drink more alcohol than before the crisis. The most notable and plausible impact is found for perceptions of the virus as a public health threat, which increased people’s propensity to buy provisions and clean the home.

Table B.2. Determinants of Private Behaviors March 24-25

	Buy Provisions	Drink Alcohol	Clean Home
Presidential Approval	0.036	0.064	-0.083*
	(0.054)	(0.055)	(0.060)
LREM Partisanship	-0.044	-1.052**	0.305
	(0.446)	(0.356)	(0.465)
Public Health Threat	0.486**	0.173	0.996**
	(0.187)	(0.172)	(0.228)
Economic Threat	0.003	-0.251	-0.241
	(0.229)	(0.207)	(0.243)
Extreme Left	-0.098	0.371	-0.434
	(0.475)	(0.492)	(0.479)
Extreme Right	0.884**	0.226	-0.182
	(0.312)	(0.320)	(0.381)
Health Risk Acceptance	-0.064	0.346**	0.074
	(0.191)	(0.201)	(0.201)
Personal Health	-0.233*	0.126	-0.100
	(0.154)	(0.148)	(0.174)
Sex	0.746**	-0.140	0.284
	(0.269)	(0.261)	(0.290)
Age	-0.026**	-0.020**	0.007
	(0.010)	(0.010)	(0.011)
Education	0.265**	0.113	-0.070
	(0.157)	(0.152)	(0.173)
Income	0.022	0.290**	-0.028
	(0.109)	(0.098)	(0.119)
Cohabits	0.028	-0.482**	0.154
	(0.322)	(0.292)	(0.333)
Employed	-0.059	0.322	-0.215
	(0.298)	(0.292)	(0.334)
Post-Speech	-0.018	0.034	0.267
	(0.265)	(0.254)	(0.282)
Constant	2.937**	0.894	2.928**
	(1.328)	(1.312)	(1.482)
Observations	774	774	774
R-squared	0.06	0.06	0.07

Note: OLS regression estimates, using respondent sampling weights. Unstandardized regression coefficients. Standard errors are in parentheses. * $p < 0.1$, ** $p < 0.05$ (one tailed)

LREM Partisanship

Given the dominance of the office of the President in the French political system, the role of the state in French politics, society, and economy, and the fact that both the presidency and parliament have been under the clear control of President Macron's LREM party for several years, examining the effect of partisanship on behavior should be straightforward. At the same time, there occasionally has been debate about the meaning and stability of partisan attachments in France, given that parties traditionally have been a mix of vehicles to support the ambitions of presidential candidates, alongside more traditional mass membership parties like the Socialist and Communist parties. Moreover, the relatively recent advent of LREM has jumbled the French party system, and it would be reasonable to assume that partisan effects may therefore be harder to demonstrate.

To examine the nature of LREM partisanship and partisan attachment in France, I therefore analyzed the stability of partisan attachments before and after the emergence of LREM, the levels of partisan attachments in France compared to other countries, and the stability of individual-level partisan attachments for LREM supporters compared to those of other parties since 2015. Table B.3 shows the proportions of respondents who reported an attachment to a political party. Importantly, given that these are panel data, these figures indicate stability at the individual level (of the same respondents over time) and thus directly reflects the impact of the founding of LREM on the same respondents over time.[1]

Table B.3. Individual-Level Stability of Partisan Attachments in France

Panel Waves	Attached	Not Attached
2015	79.5	20.5
2016	80.5	19.5
2017	79.9	20.1
2019	73.4	26.3
2020	73.6	26.4

Note: Each column entry indicates the percentage of panel respondents indicating partisan attachment.

Across 32 democracies surveyed as part of the commonly used data from the *Comparative Study of Electoral Systems* surveys (Module 4) collected between 2011 and 2016, 32.2% said they did not feel close or attached to any political party. This number ranged from 9% in Canada to 64.5% in Brazil, with a median of 37% (see also Dalton and Weldon 2007). Thus, French levels of partisan attachments compare favorably to other countries, with a lower percentage of respondents indicating that they do not feel close to a party compared to many other democracies.

Moreover, levels of individual-level partisan attachments were similar for LREM supporters compared to those of other parties.

Table B.4. Individual-Level Stability of Partisan Attachments in France

Waves	LREM	PS	Republicains	FN/RN
2015-16		88.1	91.2	85.7
2016-17		52.4	63.9	78.4
2017-19	73.7	75.8	75.9	84.8
2019-20	66.5	65.5	75.2	71
2020-20	77.7	76.7	82.1	75.6

Note: Each column entry indicates the percentage of respondents reporting the same partisan attachment from one panel wave to the next.

Results demonstrate that – despite the novelty of LREM – levels of partisanship were quite stable, especially given the upheaval in the party system. They also compare favorably to the level of partisan identification in other democracies and individual-level attachments to LREM over successive panel waves were both stable and similar to attachments to other political parties.

Do attachments to other parties matter?

Given the comparatively fragmented nature of the French party system, one additional question is whether attachment to a party other than president's party and the parliamentary majority has the opposite effect to being a government co-partisan. This question is especially relevant in light of anecdotal evidence that compliance with government restrictions during the pandemic has become a partisan issue in a number of democracies, most prominently in the U.S. Specifically, I therefore estimated models identical to those reported in Table 1, but with partisanship for each of the main opposition parties as additional independent variables. Because model results are essentially identical across specifications, I plot the coefficients from the balanced estimation model in Figure B.1. below. They show that attachments to these parties had no systematic effects on reported compliance.

Figure B.1. Effects of Partisanship on Compliance Behaviors

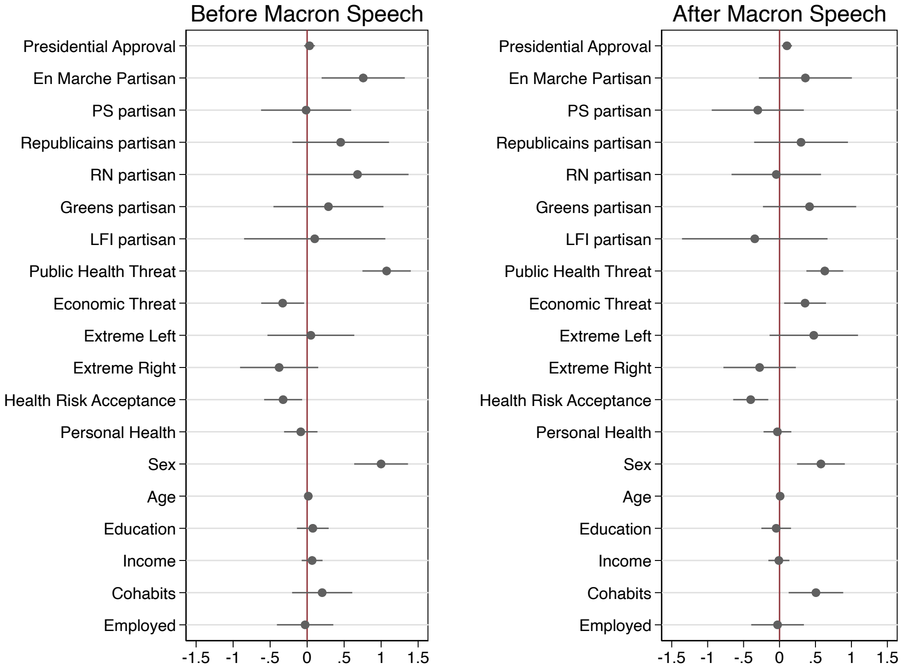

Coefficients are unstandardized coefficients from balanced logistic regression.

Notes

[1] I combined waves when there was more than one in a calendar year. Results were not affected by this.

References

Brouard, Sylvain. 2020. "Citizens' Attitudes Under COVID-19 Pandemic." Data file. https://doi.org/10.21410/7E4/EATFBW

Dalton, Russell J., and Steven Weldon. 2007. "Partisanship and Party System Institutionalization." *Party Politics* 13 (2): 179-96.

Hainmueller, Jens. 2012. "Entropy Balancing for Causal Effects: A Multivariate Reweighting Method to Produce Balanced Samples in Observational Studies." *Political Analysis* 20 (1): 25-46.
